# The Danger of Impure Drinking Water

**Published:** 1885-06

**Authors:** 


### THE DANGER OF IMPURE DRINKING WATER.

If the profession and the people need anything more to convince them of the danger of drinking impure water, it is furnished in the report made by the committee of physicians who have only recently examined the water supply of Plymouth, Pennsylvania, the city in which so much suffering and death have occurred in the past few weeks from typhoid fever.

This committee, in a report published in the *Medical News* of May 16th, says that within forty feet of the stream that supplies the reservoirs they found a patient convalescent from typhoid fever. This patient visited Philadelphia on December 25th, 1884, and while there contracted typhoid fever. He returned to his home in January and was quite ill with the disease for many weeks. On March 18th and 19th, he suffered with severe hemorrhage from the bowels.

During the course of his illness, the dejecta passed were thrown on the snow within a few feet of the stream supplying the town. These dejecta, thrown out from time to time, no doubt accumulated and remained inocuous upon the snow and frozen ground. From March 25th to March 31st, the temperature was sufficiently high to melt large quantities of snow. This thaw was followed by rain, with mild, warm weather. These thaws and rains removed the poisonous matter into the water supply. This occurred between March 25th and April 5th. The outbreak of the disease occurred between April 5th and April 15th, thus allowing from ten to fourteen days as the proper period of incubation from the time of the proven contamination of the water supply.

The opinion of the committee was strengthened by the fact that parties living on the same streets and in the same locality, who did not drink the reservoir water, but well water, did not have the fever. In one house were found two cases of the fever, in patients who had been in the habit of drinking the hydrant water from home, while other members of the house, who had not been drinking the hydrant, but the well water, were free from the disease. The committee concludes its report as follows:

1st. That the present epidemic in Plymouth is undoubtedly one of typhoid fever.

2d. That its exciting cause was the use of water from the reservoirs furnished sometime between March 25th and April 5th, and polluted by excreta from a typhoid fever patient livin g near the source of water supply.

3d. We do not believe that said epidemic was caused by drinking river water. We are led to the second conclusion from finding what, in our judgment, is sufficient evidence of past pollution of the water supply near its source, and because it is well known that excreta from *one* typhoid patient have frequently poisoned the water supply of a whole neighborhood and caused an epidemic of such fever.

### ITEMS.

Dr. F. W. McRAE, who recently graduated from the Atlanta Medical College, has located in Talbotton, Ga.

WE had a pleasant call a few days ago from Dr. J. S. Macon, a prominent physician of Hays Store, Ala.

DR. A. C. CLEMENTS, valedictorian of the graduating class of the Atlanta Medical College, session 1884–85, has located at Chauncey, Ga.

DR. J. D. WILSON, of this city, has been elected to the chair of Chemistry in the Atlanta Medical College, made vacant by the death of Dr. J. H. Logan.

FROM every portion of the State letters are coming in from members of the Association endorsing the action of that body in adopting THE ATLANTA MEDICAL AND SURGICAL JOURNAL as the official organ of the State Medical Society.

A CORRECTION.—In the minutes of the Association, as published in the last issue of THE JOURNAL, there appeared no report from the ninth district. There was a report on gynecology, made by Dr. L. G. Hardman, of Harmony Grove, and it should have so read.

DR. A. L. LOOMIS says: "A man can take two or three glasses of stimulants through the day, as he may feel an inclination, and he may continue this for perhaps twenty-five years without any harm accruing from it; but when this man reaches that period of life when the vital powers are on the decline, he suddenly feels himself growing old before his time, for he has all these years been laying the foundation for chronic endoarteritis. I believe that fifty per cent. of all diseases arise from the use of alcoholic stimulants."—*Ex.*

WE have received the first number of the *Sanitary Monitor*, a new monthly devoted to individual, family and public health, published at Richmond, Va., by J. F. Winn, M. D., editor and proprietor. We congratulate the editor upon its handsome appearance.

DEATH OF A MEDICAL CENTENARIAN.—Dr. Oliver Swaim Taylor, of Auburn, N. Y., doctor and clergyman, died recently at the age of one hundred years. He was born in New Ipswich, N. H., December 17, 1784. He graduated from Dartmouth College in 1809, and graduated in medicine in 1813. Finding teaching more congenial than the practice of his profession, he gave little attention to medicine, but taught many men who have since become famous.—*New York Medical Record.*

THE Atlanta *Journal*, one of the best evening papers published in Georgia, is making it warm for quacks in this city. It says: "The average quack doctor knows less about medicine than a street car mule, and people patronizing him ought to be labeled 'Fool.' Itinerant quacks ought to be prohibited from practicing their frauds upon the people in Georgia. The Legislature should see to it that the unwary are not robbed of their hard earnings by these tricky impostors."

EDITOR GANTT, in an editorial in the *Banner-Watchman* on the importance of an inebriate asylum in Georgia, has this to say: "Is there a tax-payer in Georgia that will begrudge his pittance to sustain an institution that will lift his neighbors from wretchedness and degradation? We trust that at the next session of our Legislature some member will undertake this great work, and secure such information and statistics as to convince that body of the entire practicability of an inebriate asylum, and we believe this is all that is necessary to secure its establishment."

SMALL-POX BY CORRESPONDENCE.—A curious but important case, in which small-pox infection was conveyed in a letter, is recorded by Mr. Karkeek, in his recent report on the sanitary condition of St. Mary Church. On March 1st, last year, a case of small-pox was reported to him in the person of a domestic servant, who had seen no one ill or recovering from small-pox, and who had not been out of the town for months. Moreover, no case of the disease had occurred in St. Mary Church or Torquay for years. On inquiry, it was found that the infected person had received letters from her sister, an inmate of the West Bromwich Small-pox Hospital, "who had unfortunately sent the germs of the disease in her letter." The case was at once removed to the Torquay Sanitarium, and the only person in the household who became ill was the recipient and reader of the letters.—*Medical and Surgical Reporter.*

THE difference between a horse thief and a medical tramp is, that the former robs to travel, while the latter travels to rob.

A PROMINENT physician in a neighboring city received the following note from a lady a few days since:

*Dear Doctor:* Please come to see my brother at once, and bring your urethra with you. Respectfully, Mrs. ——.

The doctor answered the call, and carried a catheter.

DR. W. O'DANIEL, of Bullard's, ex-President of the Medical Association of Georgia, and one of the most prominent physicians in the State, in a letter received a few days ago, says: "I am delighted to know that your JOURNAL will publish the Transactions of the Medical Association of the State. More physicians will become acquainted with the work of the Association than ever before by this arrangement."

TREASURER'S REPORT.—The following report of the Treasurer of the Medical Association of Georgia was, by some means, misplaced at the meeting in Savannah, and did not appear in the minutes of the Association published in the last issue of THE JOURNAL, as it should:

MEDICAL ASSOCIATION OF GEORGIA,
OFFICE OF TREASURER, SAVANNAH, GA., April 15, 1885.

*To the Members of the Medical Association of Georgia*—

| | |
|---|---|
| GENTLEMEN: I have collected during the year, . . . . . . . | $679 00 |
| Balance on hand last report, . . . . . . . . . . . . . . . | 126 30 |
| Total, . . . . . . . . . . . . . . . . . . . . . . . . | $805 30 |
| Disbursements during the year, as per vouchers accompanying this report, . . . . . . . . . . . . . . . . . . . . | $512 80 |
| Leaving a balance in Treasury, . . . . . . . . . . . . | $292 50 |

A large proportion of the dues are paid at the end of the year, and not at the beginning, as should be. If the members would only pay promptly when called upon at the commencement of the year, we could meet our expenses promptly, and be entirely free of debt. Allow me to urge the members to aid me in this matter.

Respectfully submitted. E. C. GOODRICH, *Treasurer.*

DR. WALTER A. CROW, of Virginia, has recently located in this city.

DR. W. S. KENDRICK, a leading physician of North Georgia, passed through Atlanta a few days since on his return from the Exposition at New Orleans.

MORE than one hundred persons were poisoned by ice cream, at a picnic party at Tallulah Falls, a few weeks since. The cream had been made about twenty-four hours when it was served. None of the cases proved fatal, though some of them were several days recovering.

TEXAS SIFTINGS tells of the way doctors collect their bills down there. Dr. Blister presented his bill of $150 to Moses Shaunberg. Moses was shocked at the size. "Vy, mine Gott," he exclaimed, "two funerals in one family would not have cost me so much as dot." "It's not too late to have a funeral yet," returned the urbane doctor, as he brought out an army size revolver. Moses took the hint. The *Medical Age* wonders how a little of that would work in this latitude.—*New York Medical Times.*

METEOROLOGICAL SUMMARY FOR THIS STATION FOR APRIL.

| | |
|---|---|
| Highest Temperature, April 25th, | 83.4 |
| Lowest Temperature, April 14th, | 35.8 |
| Greatest Daily Range of Temperature, | 28.5 |
| Least Daily Range of Temperature, | 10.0 |
| Mean Daily Range of Temperature, | 19.0 |
| Mean Daily Dew-Point, | 42.5 |
| Mean Daily Relative Humidity, | 55.0 |
| Prevailing Direction of Wind, | Northwest |
| Total Movement of Wind, | 7,439 Miles |
| Highest Velocity of Wind and Direction, | 30 Miles Northwest |
| Number of Foggy Days, | None |
| Number of Clear Days, | 11 |
| Number of Fair Days, | 12 |
| Number of Cloudy Days | 7 |
| Number of Days on which Rain or Snow fell | 10 |

